# Supplementary material for: Polyvinyl Alcohol Polymer Functionalized Graphene Oxide Decorated with Gadolinium Oxide for Sequestration of Radionuclides from Aqueous Medium: Characterization, Mechanism, and Environmental Feasibility Studies
Source: Polymers (Basel). 2021 Nov 6;13(21):3835. doi: 10.3390/polym13213835 (PMC8587516; doi:10.3390/polym13213835)
Supplement: Supplementary file 1 [file polymers-13-03835-s001.zip › polymers-1444729-supplementary.pdf]

polymers

Supporting Information

Polyvinyl Alcohol Polymer Functionalized Graphene Oxide Decorated with Gadolinium Oxide for Sequestration of Radionuclides from Aqueous Medium: Characterization, Mechanism, and Environmental Feasibility Studies

Lakshmi Prasanna Lingamdinne [1], Janardhan Reddy Koduru [1,*], Yoon-Young Chang [1], Mu. Naushad [2] and Jae-Kyu Yang [1,*]

[1] Department of Environmental Engineering, Kwangwoon University, Seoul 01897, Korea; swethasiri86@gmail.com (L.P.L.); yychang@kw.ac.kr (Y.-Y.C.)
[2] Department of Chemistry, College of Science, King Saud University, P.O. Box 2455, Riyadh 11451, Saudi Arabia; mnaushad@ksu.edu.sa
* Correspondence: reddyjchem@gmail.com (J.R.K.); jkyang@kw.ac.kr (J.-K.Y.); Tel.: +82-02-9405496

1.1. Analytical Instruments

X-ray powder diffraction (XRD) analysis of the nanocomposites was carried out using Rigaku D/Max–2500 X-ray diffractometer (Tokyo, Japan). Spectrum GX-Fourier Transform Infrared Spectroscopy (FT-IR) (Perkin-Elmer, Waltham, MA, USA) was used for functional groups analysis of the GO-Gd and PGO-Gd. IFS 66/S, FRA106/S (BRUKER OPTICKGMBH, USA) was used for FT-Raman spectrum analysis. Thermo scientific ESCALAB–210 (Spain) X-ray photoelectron spectroscopy (XPS) was used for elemental composition analysis. S–4300 & EDX–350 (Hitachi, Japan) was used for measuring the surface morphology of samples. HR-TEM (JEM-4010, JEOL, and Peabody, MA, USA) was used to measure the shape and particle size of the adsorbents. Autosorb-1, Quanta chrome instrument (Boynton Beach, FL, USA) was used to measure the Brunauer, Emmett and Teller (BET) surface area and pore sizes of samples. TGA instruments (SDT 2960, USA) was used to thermal gravimetric analysis of composite. The residual U(VI) and Th(IV) concentration was determined using Optima 2100 DV inductive coupled plasma-optical emission spectroscopy (ICP–OES) (Perkin-Elmer, Waltham, MA, USA) equipped with an auto sampler.

1.2. Chemicals

All the chemicals used were of analytical reagent grade. Deionized double distilled water was used throughout the experimental studies. Graphite flake powder and $Gd(NO_3)_2$ 6H2O was supplied by Sigma Aldrich (USA).Poly vinyl alcohol, H_2SO_4 (98%), HCl (40%) and NH_4OH (56.6%) were supplied by the Samchun Pure Chemicals Co. Ltd. (Korea). $KMnO_4$ and NaOH (98%) were supplied by Kanto Chemical Co. Inc. (Japan). $NaNO_3$ (98%) was supplied by Duksan Pure Chemicals (Korea). H_2O_2 (30%) was supplied by Junsei Chemicals Co. Ltd. (Japan).

Table S1. Physico and chemical properties of Ground water.

SO_4^{2-}	PO_4^{3-}	NO_3^-	HCO_3^-	Cl^-	Na+	Ca^{2+}
53.2	N/A	15.7	172	19.6	8.406	68.32

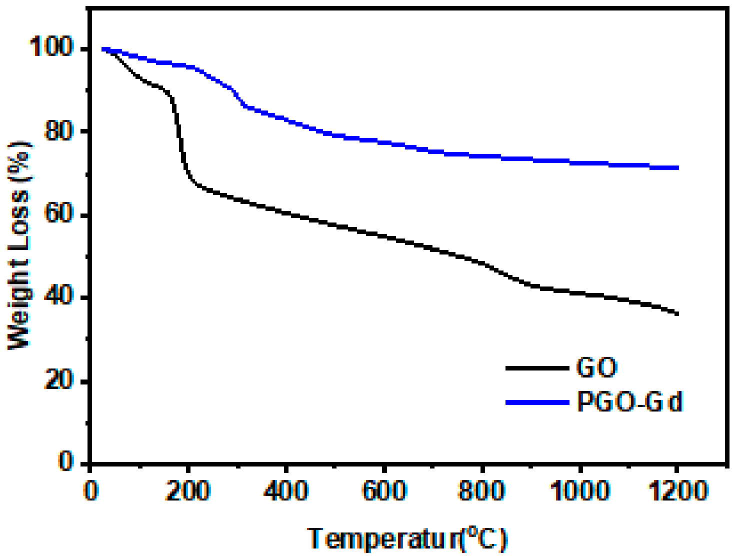

Figure S1. TGA of GO and PGO-Gd.

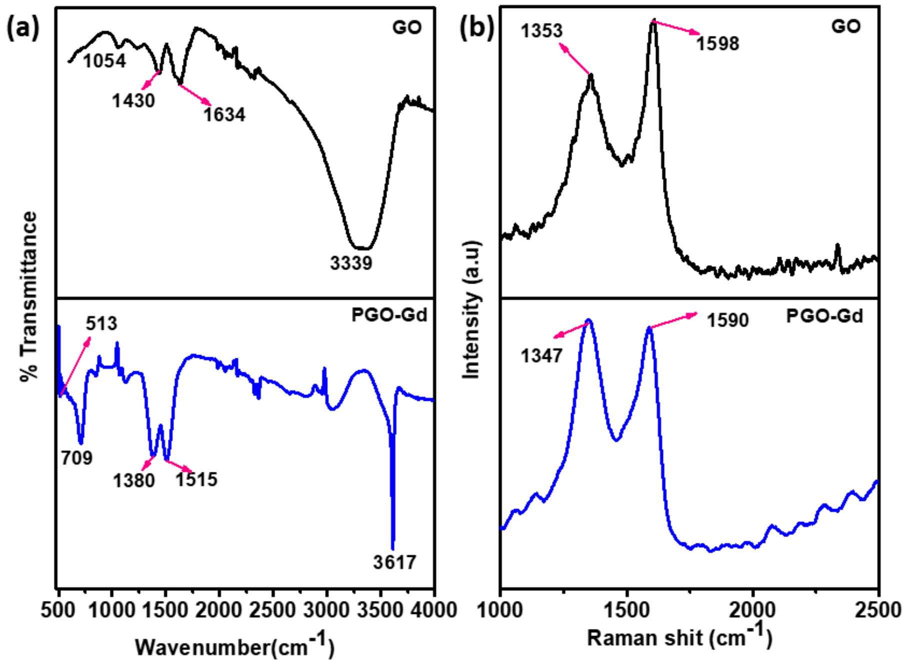

Figure S2. FT-IR (**a**), and Raman spectroscopy (**b**) of GO and PGO-Gd.

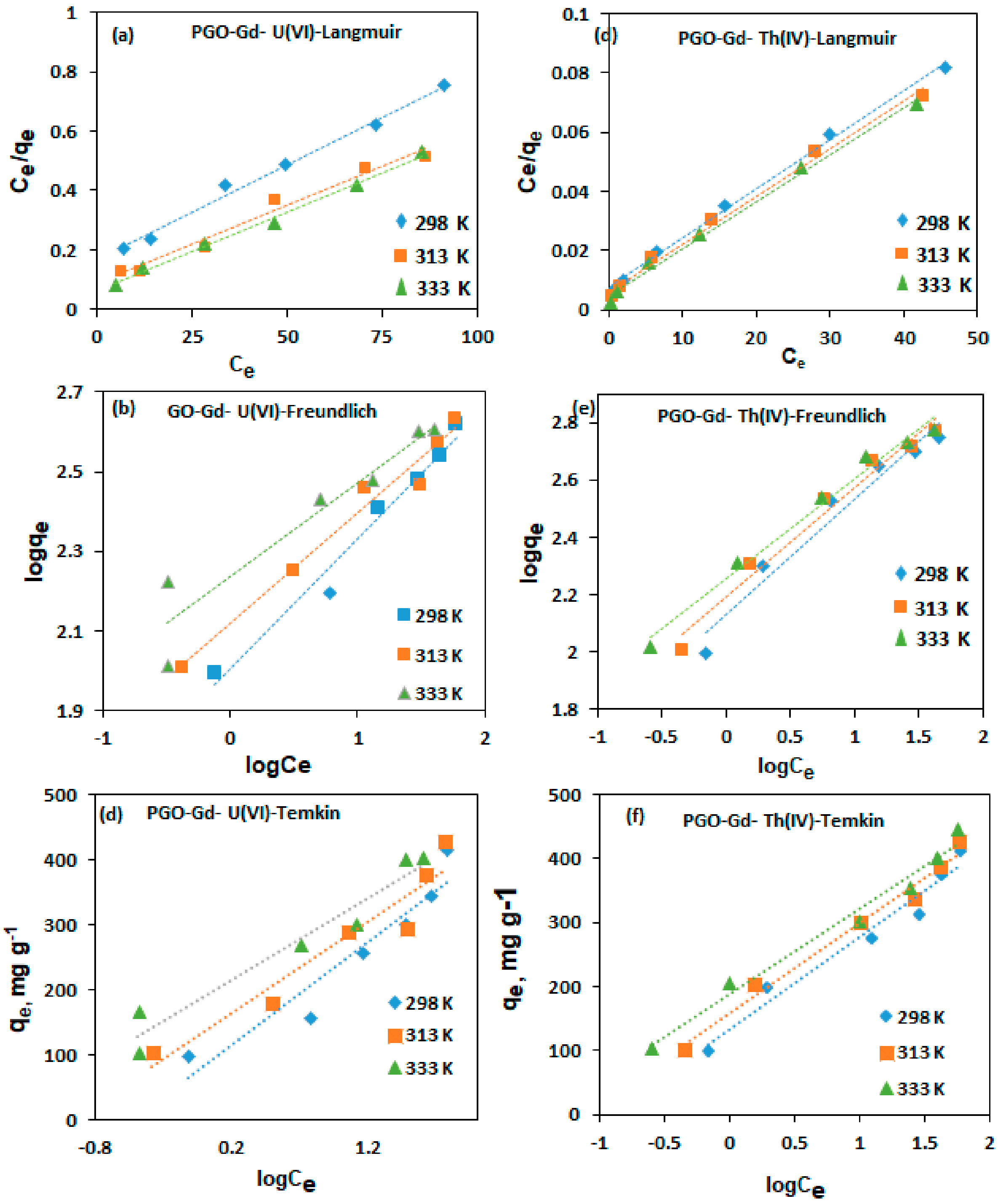

Figure S3. Isotherms of U(VI) and Th(IV) onto PGO-Gd (0.1g/L,pH-4 and intial concentration 10-100 mg/L).

Table S2. Thermodynamic parameters for the U (VI) and Th (IV) on GO-Gd and PGO-Gd at pH 4.

Metal ion	Temperature, K	ΔG° (Kj mol^{-1})	ΔH° (kJ mol^{-1})	ΔS° (kj mol $^{-1}$)
	298	-5.04984334		
U(VI)	313	-5.85923678	10.204	0.0509
	333	-6.7870081		
	298	-5.17095723		
Th(IV)	313	-6.03404882	9.6641	0.0495
	333	-7.05718225		

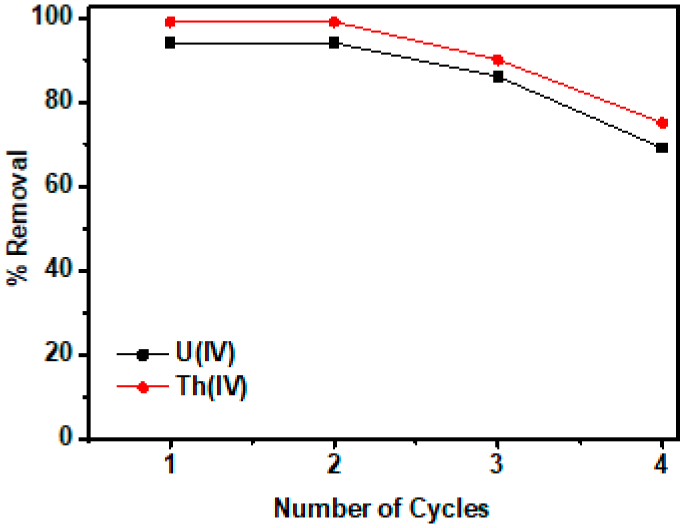

Figure S4. Recyclable studies U(VI) and Th(IV) onto PGO-Gd .
